# Response of deep soil moisture to different vegetation types in the Loess Plateau of northern Shannxi, China

**DOI:** 10.1038/s41598-021-94758-5

**Published:** 2021-07-23

**Authors:** Qingping Gou, Qingke Zhu

**Affiliations:** 1 School of Soil and Water Conservation, Beijing Forestry University, Beijing, 100083 China; 2 Engineering Research Center of Forestry Ecological Engineering, Ministry of Education, Beijing Forestry University, Beijing, 100083 China

**Keywords:** Hydrology, Forest ecology

## Abstract

Deep soil moisture is a highly important source of water for vegetation in the semiarid Loess Plateau of China, vegetation restoration reduced the deep soil moisture, but how to better quantify the impact of vegetation restoration on deep soil moisture is lack of certain understanding. To explore the impact exerted by different types of vegetation on deep layers of the soil moisture, the 0–10 m soil moisture content profile was measured before and after the rainy season in *Armeniaca sibirica, Robinia pseudoacacia, Populus simonii, Pinus tabuliformis, Hippophae rhamnoides* and in natural grassland in Wuqi County in Shannxi Province. These results showed that the highest soil moisture in the shallow layers (0–200 cm) was exhibited in the *P. simonii* forest, which was followed by that in the natural grassland. Both of these results were significantly higher than that those of the *A. sibirica, P. tabuliformis, H. rhamnoides* and *R. pseudoacacia* forests. The soil moisture in the deep layer (200–1000 cm) of the natural grassland was significantly higher than that of the other vegetation types. The annual precipitation that recharges the depth of soil moisture was the highest in natural grassland and the lowest in *P. simonii*. The inter-annual soil moisture replenishment is primarily affected by rainfall and vegetation types. Compared with the natural grassland, the *CSWSD* (the comparison of the soil moisture storage deficit) of different vegetation types varies. In the shallow soil layer, *P. simonii* is the lowest, and *R. pseudoacacia* is the highest. In the deep soil layer, *R. pseudoacacia* and *P. simonii* are the highest; *H. rhamnoides* is the second highest, and *A. sibirica* and *P. tabuliformis* are the lowest. These results indicate that vegetation restoration can significantly reduce the amount of water in the deep layers of the soil. In the future vegetation restoration, we suggest emphasizing natural development more strongly, since it can better maintain the local vegetation stability and soil moisture balance.

## Introduction

Soil water is a primary limiting factor for vegetation rehabilitation in arid and semi-arid regions^1,2^. It plays an important role in the water cycle of the terrestrial ecosystem. In particular, soil moisture directly or indirectly participates in the processes of runoff generation, soil evaporation and plant transpiration^1,3,4^. Approximately 100 m of thick loose porous loess covers the Chinese Loess Plateau and functions as a soil reservoir, which can effectively alleviate drought and provide sufficient water for plant growth^5–7^. Moreover, the groundwater is usually less than 30 to 100 m above the surface of this area^8^, and very little of the groundwater can be used by soil evaporation and vegetation transpiration^9^. Therefore, the soil moisture stored in the soil profile, particularly in the deep soil, plays a vital role in the stable growth of plants^9–11^.

Since 1999, China has implemented the project of “the Grain for Green Program” to control soil erosion and curb ecological deterioration^12,13^. As a result of this project, the vegetation coverage of the Loess Plateau in China increased from 31.6% in 1999 to 59.6% in 2013, and the annual sediment discharge in the Yellow River dropped to its lowest level in history, approximately 0.2 Gt^14^. Vegetation restoration had made a substantial contribution, while many scholars have simultaneously evaluated the impact of vegetation restoration on the ecosystem services^15,16^. Examples include an increase in carbon sequestration by the ecosystem^16^ and a decrease in soil water^17^. In particular, there is a substantial consensus about the impact of the project on the hydrological cycle. It is well known that a large amount of soil water is required for the successful implementation of this project. Land use changes affect the dynamics of soil water^11,18–20^, water balances^21–23^ and other eco-hydrological processes^6^. However, owing to the unique climate conditions of “low precipitation and high evaporation” in the Loess Plateau, limited water resources make it difficult to maintain the current vegetation restoration currently conducted in the Loess Plateau. Therefore, the excessive depletion of deep soil water resources and the lack of long-term precipitation supplementation lead to the formation of a dry soil layer, which has become a very serious problem in the Loess Plateau^24,25^. Currently, it is highly important to study the dynamic impact of different vegetation types on soil moisture, the long-term rational management of water resources and land use planning, and the solution of unsustainable vegetation restoration. The relationship between vegetation types and soil moisture has become a hot issue in ecohydrology. It has been widely discussed, and stakeholders have made a substantial amount of progress.

The effects of different vegetation types on soil moisture have been widely studied. The seasonal variation and vertical distribution of soil moisture under different land use patterns were studied in the Loess Plateau of China^26,27^. The results show that there are three main changes of soil moisture: an increase, a decrease and fluctuation^28^. The vertical distribution of soil moisture differed in different seasons, and surface soil moisture was strongly affected by the soil texture and rainfall^29^. The loss of soil water primarily occurs during the growing season of vegetation, but precipitation in the rainy season cannot completely supplement the loss of soil water, thus, restraining the sustainability of vegetation restoration in the Loess Plateau of China^5^. Mei et al.^30^ studied the effects of vegetation types on soil moisture and the effects of inter-annual changes in the Loess Plateau. Liu et al.^31^ found that rainfall replenishment of soil water can barely exceed the depth of 2 m soil layer. In Southern Italy, Longobardi^32^ studied the soil moisture temporal and variations of a perennial lawn grassland and found that the annual soil water cycle was significantly affected by the fluctuation of precipitation. Many researchers have also studied the soil water deficit of different sites of vegetation restoration^33–35^ and showed that the large-scale vegetation restoration in the Loess Plateau has caused different degrees of a decline in soil moisture, resulting in a soil water deficit for different vegetation. However, there are few studies on the profile distribution and interannual variation of soil moisture under different types of vegetation restoration, particularly the quantification of soil water deficit in different types of vegetation.

To explore the response of the soil moisture to vegetation in the Loess Plateau, China, many studies have indicated that compared with grassland and farmland, woodland will cause a soil moisture deficit. In view of the problem of soil water deficit, some related researchers proposed to use relative soil water deficit to evaluate the impact of different types of vegetation on ecological hydrology. Taking the local natural grassland or cropland as the background value, such as the depletion of soil water storage^26^, compared soil water deficit index^36^, etc. However, most of these studies focused on shallow soil moisture^17,37,38^, moreover, there are relatively few reports on quantifying the deep soil water deficit of different vegetation types with the comparison of the soil moisture storage deficit (CSWSD). Based on different types of vegetation in the Jinfoping Watershed in the loess area of northern Shaanxi, this study focused on the effects utilized by different types of plant rehabilitation on the soil moisture.

This paper attempts to answer three questions: First, the characteristics of the distribution and variation of the vertical section of soil moisture different types of vegetation; second, inter-annual changes of soil water storage under different vegetation types; third, the soil moisture deficit of different types. We hypothesized that natural grassland has no soil water deficit, and arbors and shrubs will consume deep soil moisture, resulting in a soil moisture deficit.

## Materials and methods

### Study sites

The study area is located in the Jinfoping Catchment of Wuqi County, Yan'an City, Shaanxi Province, China (36°33′33″—37°24′27″N, 107°38′57″—108°32′49″E). The elevations range from 1233 to 1809 m above sea level and are typical loess hilly and gully areas. The climate type is a temperate continental monsoon climate, with an annual average temperature of 7.8 °C and a frost-free period of 120–155 days. The average annual rainfall of the county is 478.3 mm, which accounts for 62.4% of the total annual precipitation that falls during July to September with heavy rain. The annual potential evaporation ranges from 400 to 450 mm. The soil is primarily Huangmian soil (Calcaric Cambisols) (IUSS Working Group WRB, 2015) that has 20.9% field water holding capacity and 4.7% wilting moisture. Since the implementation of the return of the farmlands to forests in 1998, the restoration of the vegetation in Wuqi County has had substantial effects.

### Experimental site design

In this study, Siberian apricot (*Armeniaca sibirica Lam.)* (AS), Chinese pine (Simon*Pinus tabuliformis Carr.)* (PT), Simon poplar (*Populus simonii Carr.)* (PS), Black locust (*Robinia pseudoacacia Lam.)* (RP), Sea buckthorn (*Hippophae rhamnoides Lam.)* (HR) and natural grassland (NG), the primary types of vegetation after vegetation rehabilitation in the Jinfoping Catchment, were selected as the research objects, with the natural grassland that mainly includes *Stipa capillata* and *Poa annual* as the control group. To make the experiment comparable, the direction of the slope and the gradient of each vegetation type were basically the same. Sample plots of 20 m × 20 m were set up in the arboreal forest, 5 m × 5 m in the shrub forest and 1 m × 1 m in the grassland to investigate and record the growth the vegetation. All the vegetation types are not irrigated. A sample plot was established for each vegetation type, and a total of six sample plots were established in this study. The results are shown in Table 1.

**Table 1:** Basic situation of the different types of vegetation.

Vegetation characteristics	AS	PT	HR	RP	PS	NG
Altitude/m	1410	1420	1425	1410	1434	1431
Slope gradient/°	23	25	25	20	24	18
Slope position	Up-middle	Up-middle	Up-middle	Up-middle	Up	Up
Slope aspect	East	East	Southeast	Northeast	East	Southeast
Average high/m	3.10	3.20	2.50	10.90	8.90	
Average DBH/cm	2.60	2.20	1.80	8.60	8.60	
Canopy density/%	40%	60%	90%	80%	85%	
Planting density	2 × 3	2 × 3	1 × 1	2 × 3	2 × 3	

### Data collection

**Figure 1:**
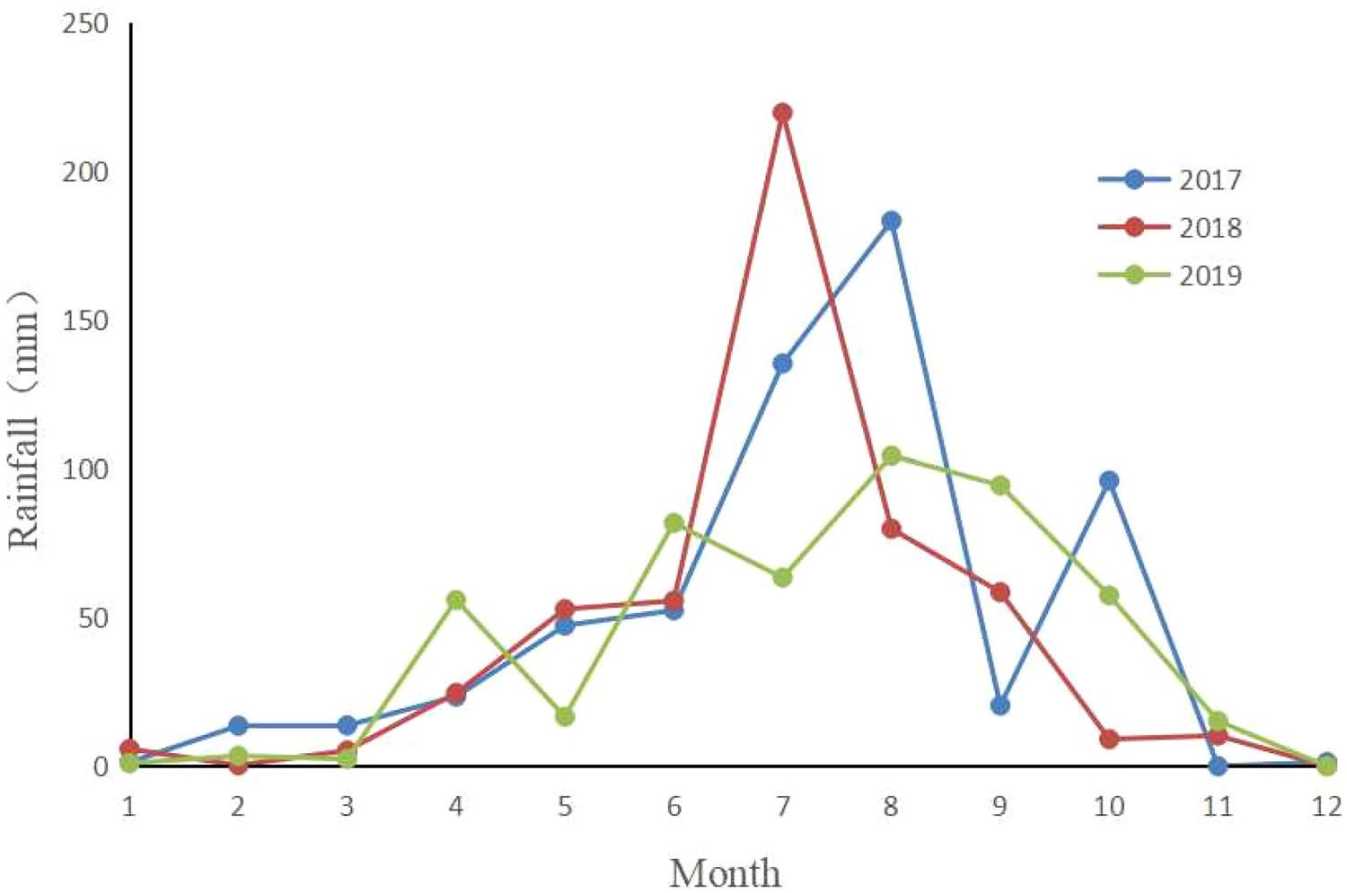
Distribution of the monthly precipitation in 2017–2019.

Three points were randomly selected from each plot and sampled with an artificial soil drill. After each point was taken, it was immediately packed into an aluminum box with a diameter of 5 cm and brought back to the laboratory to measure the gravimetric soil moisture using a oven-drying method (105 °C, 24 h)^39^. The soil moisture sampling depth of all the types of vegetation was 10 m, 0–1 m sampled at intervals of 10 cm, and 1–10 m sampled at intervals of 20 cm. Three soil samples were repeatedly taken from each layer of each sample point, and the average value of the three soil samples was taken as the soil moisture content value of the soil layer. No rain was observed for 7 days before each sampling. The rainfall data came from the China meteorological data sharing network (Fig. 1).

The experiment was conducted from October 25 to November 10 in 2017 (after the rainy season) and two periods in 2018, from May 25 to May 30 (before the rainy season) and from October 3 to October 11 (after the rainy season). In addition, the experiment was conducted for two periods from May 1 to May 10 and from October 5 to October 12 in 2019.

To facilitate the calculation of the soil water storage, 1 m of the soil profile was excavated with a spade in each sample plot, and undisturbed soil samples were taken every 20 cm using a ring knife. Three replicates were sampled from each layer, and a total of 5 layers were sampled. The soil samples were taken back to the laboratory and the bulk density of the soil was determined using the oven-drying method. The average values of the three replicates were used as the soil bulk density values. The soil bulk density distribution in 1 m profile of each vegetation type is shown in Table 2.

**Table 2:** Average bulk density at the 0–100 cm depth (mean ± SD).

Soil depth/cm	AS	PS	HR	PT	NG	RP
0–10	1.198 ± 0.011	1.244 ± 0.054	1.242 ± 0.007	1.084 ± 0.065	1.208 ± 0.012	1.084 ± 0.065
10–20	1.210 ± 0.058	1.251 ± 0.033	1.262 ± 0.018	1.151 ± 0.023	1.243 ± 0.035	1.270 ± 0.071
20–30	1.172 ± 0.042	1.222 ± 0.024	1.232 ± 0.007	1.202 ± 0.005	1.260 ± 0.005	1.202 ± 0.005
30–40	1.160 ± 0.028	1.278 ± 0.019	1.215 ± 0.051	1.192 ± 0.014	1.283 ± 0.016	1.210 ± 0.077
40–50	1.272 ± 0.003	1.259 ± 0.010	1.225 ± 0.005	1.225 ± 0.015	1.280 ± 0.000	1.225 ± 0.015
50–60	1.295 ± 0.025	1.248 ± 0.017	1.233 ± 0.028	1.243 ± 0.019	1.287 ± 0.032	1.251 ± 0.005
60–70	1.318 ± 0.007	1.218 ± 0.107	1.238 ± 0.013	1.274 ± 0.010	1.276 ± 0.006	1.274 ± 0.010
70–80	1.322 ± 0.034	1.196 ± 0.016	1.236 ± 0.001	1.285 ± 0.017	1.304 ± 0.006	1.293 ± 0.065
80–90	1.325 ± 0.024	1.325 ± 0.012	1.301 ± 0.004	1.295 ± 0.012	1.304 ± 0.005	1.298 ± 0.018
90–100	1.318 ± 0.008	1.301 ± 0.035	1.302 ± 0.010	1.304 ± 0.034	1.301 ± 0.012	1.307 ± 0.061

### Method of analysis

Soil moisture storage is calculated using the following formula (Mei et al. ^41^).

$$Q_{i} = \frac{{10 \times \theta_{i} \times BD_{i} \times H}}{\rho }(i = 1,2,3, \ldots ,n)$$

where *Q*_*i*_ is the soil moisture storage (mm); *θ*_*i*_ is the gravimetric soil moisture content (g/g); H is the thickness of soil layer (cm); *ρ* is the water density (1 g/cm^3^), and *BD*_*i*_ is the bulk density (g/cm^3^); *i* represents the soil sequence, and n is the number of layers that were measured. When calculating the soil water storage, the measured values above the depth of 1 m and the measured values below 1 m were used to calculate the soil bulk density at 1 m. The relevant studies showed that the soil bulk density barely changes under 1 m of the soil profile^40^. In addition, it is difficult to sample undisturbed soils below a depth of 1 m^41^.

The inter-annual change in the soil water storage indicates:

$$\Delta Q_{i} = Q_{i} 2 - Q_{i} 1$$

where *Q*_*i*_2 represents the final value of soil water storage (mm) that was measured in October; *Q*_*i*_1 represents the initial value of the soil water storage (mm) that was measured in May. The rainfall from May to October comprised 91%, 91% and 84% of the annual total rainfall from 2017 to 2019, respectively (Fig. 1). Thus, having *ΔQ*_*i*_ represent the change of the soil water storage within the raining season is a logical approach.

Natural grassland is formed naturally without human disturbance, and its soil moisture can reflect the background value before artificial afforestation. Therefore, taking the natural grassland as a compare. A new indicator called the comparison of the soil moisture storage deficit (*CSWSD*) is proposed to quantify soil water deficit of different vegetation types^36^.

The soil water deficit is expressed by the CSWSD:

$$CSWSD = \frac{1}{k} \times \sum\limits_{i = 1}^{k} {\frac{{Q_{ci} - Q_{i} }}{{Q_{ci} - Q_{m} }}}$$

where *CSWSD* indicates the comparison of the soil moisture storage deficit; *Qi* is the plot of soil moisture storage in the soil layer i. *Q*_*ci*_ is the comparison of the plot soil moisture storage that is owing to natural grassland soil moisture storage in this study. *Q*_*m*_ represents the soil moisture storage in the wilting point.* k* is the number of soil layers. If the *CSWSD* > 0, it indicates that the soil water in the forestland is in a state of relative deficit, which indicates that vegetation could accelerate water consumption. If *CSWSD* < 0, it indicates that the soil water in the forestland is in a state of relative reserve.

Microsoft Excel 2016 was used to calculate the average soil water content and the standard errors of different vegetation types. A univariate analysis of variance (ANOVA) and multiple comparisons (LSD) were used to analyze the significant difference of the soil water content between the different vegetation types in Microsoft Excel 2016 and SPSS 17.0(SPSS, Inc., Chicago, IL, USA).

## Results and analysis

### Distribution characteristics of the vertical profile of soil moisture in different vegetation types

**Figure 2:**
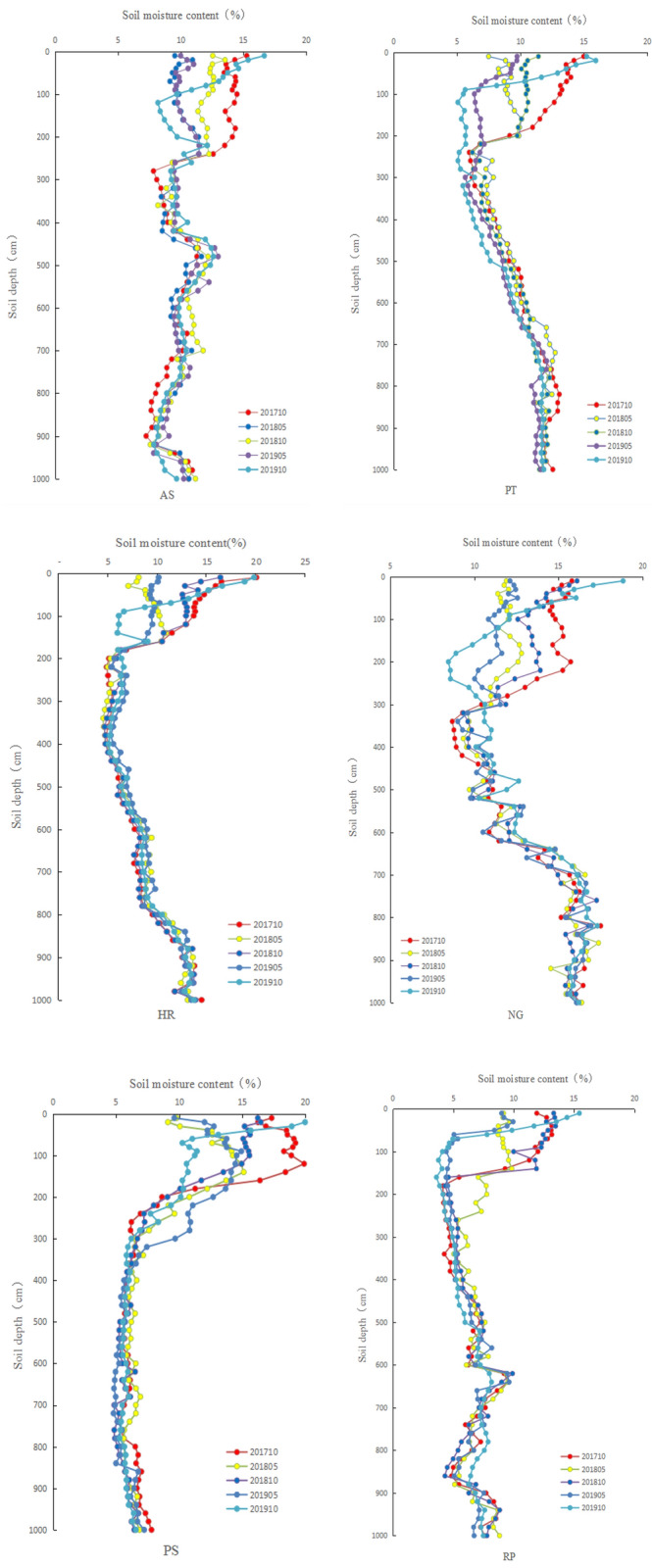
Distribution of the soil moisture in different types of vegetation at 0–10 m.

The soil moisture in the vertical profiles of different types of vegetation is shown in Fig. 2. The figure shows that the soil moisture of AS fluctuates with the depth of the soil layer, while the soil moisture of the PT, HR and NG first decreased and then increased with the depth of the soil layer. The soil moisture of the PS decreased first and then remained stable with the depth of the soil. The variation of the soil moisture of the RP the soil depth was more difficult to detect. The shallow soil moisture of each vegetation type varied greatly in different periods, while the deep soil moisture was relatively stable. The shallow soil moisture of each type of vegetation in October 2017 was significantly higher than that of the other soil moisture survey periods.

### Average soil moisture variation of the different vegetation types

Whether in a shallow layer (0–200 cm) or a deep layer (200–1000 cm), the average soil moisture of the NG differs significantly from that of the other types of vegetation. The soil moisture of the PS was the highest (15.07%) in the shallow layer (0–200 cm), while the average soil moisture of the RP was the lowest (9.02%), which differed significantly from that of the other types of vegetation. There was no significant difference in the average soil moisture among the AS, PT and HR. In the deep layer (200–1000 cm), the average soil moisture of the NG was significantly higher than that of the other vegetation types, while the RP and PS had the lowest amount of soil moisture, 6.44% and 6.13% respectively. The standard deviation can reflect the heterogeneity of the soil moisture in the profile. The standard deviation of the HR was the largest among all the types of vegetation. Therefore, the distribution of soil moisture for the HP has the largest degree of variation (Table 3).

**Table 3:** Variance analysis and multiple comparisons of average soil moisture (%) in different soil layers of different types of vegetation (0–200 cm and 200–1000 cm).

Soil depth(cm)	0–200				200–1000			
Vegetation types	Mean ± SD	Min	Max	CV	Mean ± SD	Min	Max	CV
AS	13.00 ± 1.47b	10.96	15.96	0.11	9.73 ± 1.25b	7.72	12.82	0.13
PT	11.15 ± 2.68b	7.40	15.10	0.24	9.55 ± 2.39b	5.56	12.39	0.25
HR	12.17 ± 4.08b	5.81	20.01	0.34	8.41 ± 2.87c	5.08	14.19	0.34
RP	9.02 ± 3.35c	4.02	13.68	0.37	6.44 ± 1.27d	4.25	8.86	0.20
RS	15.07 ± 2.59a	9.38	19.26	0.17	6.13 ± 0.67d	5.35	8.80	0.11
NG	14.12 ± 1.59ab	11.93	17.32	0.11	13.34 ± 2.66a	9.50	17.41	0.20

One-way analysis (α = 0.05, LSD). All numbers followed by the same letters in the same column are not significantly different, while those marked with different letters are significantly different.

### Soil water inter-annual change of different vegetation types

Through the observation and analysis of the soil moisture content before and after rainfall, the intersection point of the soil water content measured twice is the depletion or recharge depth, and the difference of the twice the soil water storage measured is the soil water consumption or recharge (mm)^42,43^. From May 2018 to October 2018, with the exception of the PT (29 mm), the other types of vegetation had the largest amount of soil water recharge, of which NG and AS was the largest (58 mm). From May 2019 to October 2019, the soil water recharge of the HR reached a maximum of 51 mm, and that of the RP reached its lowest of 27 mm. In general, the depth of soil moisture recharged with different vegetation types in 2018 was greater than that in 2019, which may be because the annual rainfall in 2018 (521.6 mm) was greater than that in 2019 (495.9 mm). The maximum depth of soil moisture recharge took place in the NG, while the PS had the smallest depth (Table 4).

**Table 4:** The recharge depth (cm) and amount (mm) of soil moisture storage in different vegetation types.

Different periods	AS	PT	HR	RP	PS	NG
2018.05–2018.10	240/58	140/28	120/63	140/53	120/41	300/58
2019.05–2019.10	100/42	90/37	70/51	70/25	50/37	120/38

### A comparison of the soil moisture storage deficit of different vegetation types

**Figure 3:**
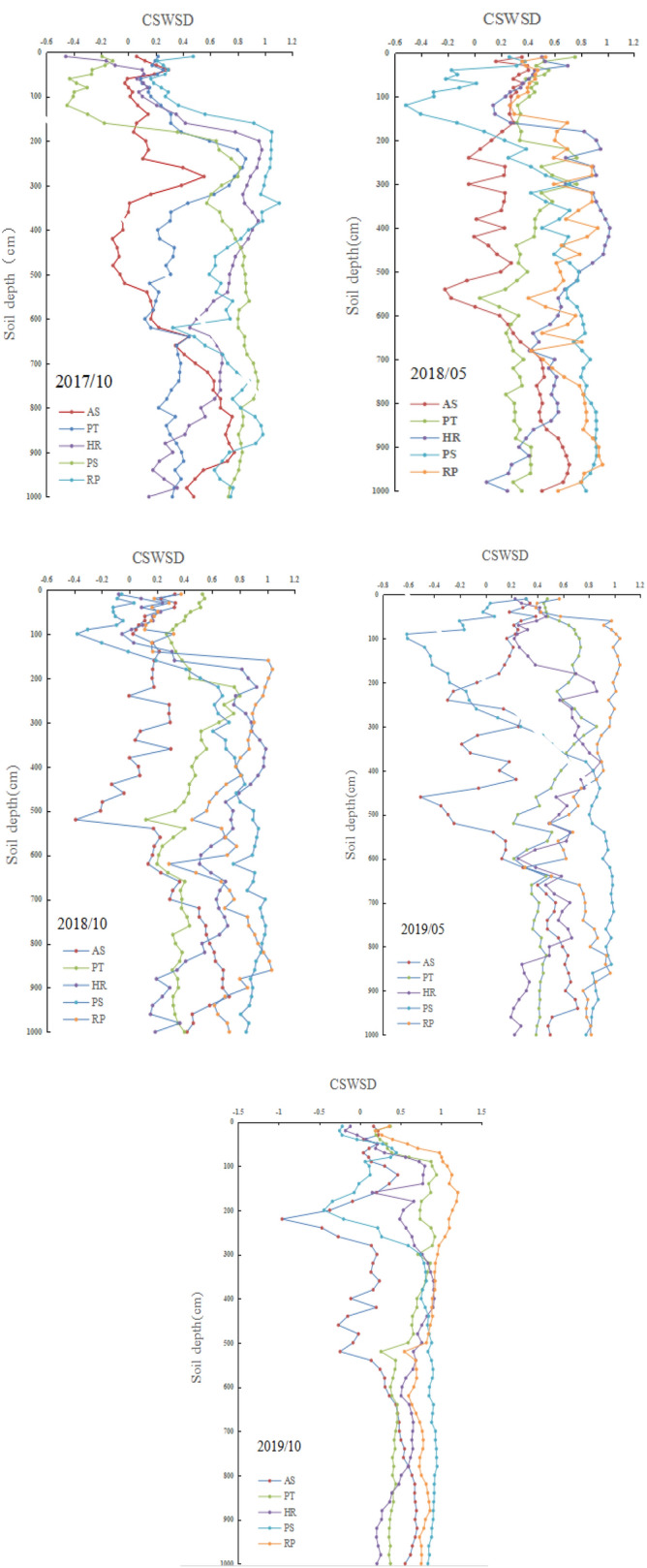
The comparison of soil moisture storage deficit of different vegetation types.

The compared soil moisture storage deficit (*CSWSD*) of different vegetation types can indicate the degree of soil water deficit. As shown in Fig. 3, the *CSWSD* of RP was the highest in different years, followed by the PS, HR in the middle and AS as the lowest. It can also be seen from Fig. 3 that the *CSWSD* in May is generally more serious than that in October. This indicates that soil moisture will be recharged for a certain extent in October after the influence of rainy season for a year. The *CSWSD* was negative in the soil layers. For example, in October 2017, the AS was 400–500 cm. This indicates that compared with the NG, there is no deficiency of AS in this soil layer.

A different type of analysis was used to facilitate the study of soil water deficit. Therefore, the soil moisture of 0–1,000 cm was divided into a shallow layer (0–200 cm) and a deep layer (200–1,000 cm) for ANOVA and multiple comparisons. As can be seen from Table 5, the CSWSD (0–200 cm) of the different vegetation is significantly altered, among which, PS had the lowest CSWSD, while RP had the highest. For the same vegetation, the CSWSD was greater in May than in October. It can be seen from Table 6 that the CSWSD (200–1,000 cm) of different vegetation is significantly altered. RP and PS had the most serious deficit, followed by HR, PT and AS. There was no obvious regularity of the CSWSD from year to year.

**Table 5:** The CSWSD of different types (0–200 cm).

Time	AS	PT	HR	PS	RP
2017/10	0.09 ± 0.09b	0.24 ± 0.13b	0.18 ± 0.35b	− 0.19 ± 0.30c	0.44 ± 0.31a
2018/05	0.29 ± 0.08b	0.44 ± 0.12a	0.44 ± 0.23a	− 0.07 ± 0.27c	0.45 ± 0.12a
2018/10	0.18 ± 0.10c	0.41 ± 0.09a	0.22 ± 0.28bc	− 0.02 ± 0.24d	0.37 ± 0.35ab
2019/05	0.21 ± 0.11d	0.61 ± 0.11b	0.38 ± 0.18c	− 0.22 ± 0.27e	0.82 ± 0.26a
2019/10	0.14 ± 0.20 cd	0.57 ± 0.29b	0.36 ± 0.35bc	0.01 ± 0.28d	0.83 ± 0.37a

Different letters on the same line indicate significant differences (*p* < 0.05).

**Table 6:** The CSWSD of different types of vegetation (200–1000 cm).

Time	AS	PT	HR	PS	RP
2017/10	0.33 ± 0.30c	0.38 ± 0.19c	0.64 ± 0.23b	0.81 ± 0.09a	0.80 ± 0.17a
2018/05	0.31 ± 0.26b	0.38 ± 0.15b	0.65 ± 0.24a	0.74 ± 0.17a	0.74 ± 0.15a
2018/10	0.28 ± 0.28d	0.41 ± 0.15c	0.64 ± 0.25b	0.84 ± 0.11a	0.77 ± 0.16a
2019/05	0.26 ± 0.34c	0.47 ± 0.15b	0.54 ± 0.20b	0.75 ± 0.32a	0.79 ± 0.15a
2019/10	0.28 ± 0.38c	0.54 ± 0.19b	0.59 ± 0.21b	0.80 ± 0.22a	0.81 ± 0.13a

Different letters on the same line indicate significant differences (*p* < 0.05).

## Discussion

### Effects of the different types of vegetation rehabilitation on soil moisture

In recent years, some studies have shown that during the process of vegetation restoration in the Loess Plateau, different types of vegetation have caused a soil water deficit, forming a drying soil layer^25,33^. However, under the same topographic conditions, there are few reports on the 0–10 m soil moisture of the different types of vegetation. In our study, different types of vegetation varied in their degree of distribution in the profile. Compared with the grassland, the deep soil moisture of the RP and PS was seriously deficient, and that of the AS, HR and PT was also deficient to varying degrees (Fig. 3, Table 6). This may be influenced by the tree age, tree size and coverage of the vegetation^44,45^.

The shallow soil moisture of the different vegetation types in October (after the rainy season) was greater than in May (before the rainy season). The primary reason is that after the rainy season, the soil moisture is recharged by rainfall. The results are consistent with the findings from previous studies^5,39^. In this study, the soil moisture of the shallow (0–200 cm) PS was significantly higher than that of the other types of vegetation, which may be due to the heavy litter that has a significant effect on the interception of rainfall. The shallow soil moisture in October 2017 was significantly higher than that in the other survey periods, primarily because the rainfall from May to October 2017 was 534.6 mm (Fig. 1), which was much higher than the multi-year average rainfall of 467.8 mm^10^. Although a substantial amount of the soil water was recharged during this period, the consumption of the soil water was also large from October 2017 to May 2018. This may be due to the redistribution process of the rainfall infiltration in the soil layer that will last for a period of time, while the soil evaporation has been continuing.

However, rainfall is the only source of the soil water recharge in the Chinese Loess Plateau. It is difficult to recover the dried soil layer, particularly the deep soil layer, caused by the deficiency of soil water. The primary reason is that the depth of the soil water supply by rainfall is limited. In normal years of precipitation, the depth of the soil water recharge by precipitation is usually 1 m. In extremely wet years, the recharge of the soil moisture by precipitation will reach 2 m^31^. In this study, the maximum soil moisture that was replenished was in NG (300 cm) in 2018. The water recharge depth of other vegetation types is typically 100 cm. The replenishment depth in 2018 was also generally higher than in 2019, primarily because the rainfall in 2018 (521.6 mm) was higher than in 2019 (495.9 mm). However, the deep soil moisture changed slightly. Compared with the NG, the CSWSD in the deep soil layer (200–1000 cm) was greater than 0, indicating that the deep soil moisture was deficient to some extent. Cheng and Liu^45^ used long-term positioning observation to show that the variation of soil moisture in different land use types was primarily affected by the annual rainfall and evapotranspiration of the vegetation. Woodland evapotranspiration is substantial, and more soil water will be used in the process of woodland evapotranspiration. In dry years, deep soil moisture provides essential water for plant growth. In recent decades, the rainfall in the Chinese Loess Plateau decreased and the temperature increased^46^. This warming and drying has increased the use of the deep soil moisture by vegetation, resulting in the deficiency of deep soil moisture, while the depth of the rainfall recharge is limited, resulting in the drying of the soil layer and deteriorating the local ecological environment. In conclusion, CSESD can better reflect the soil moisture deficit of different vegetation types. It can not only effectively eliminate of local soil moisture background value, but also be directly used for comparison between different vegetation types.

### Insights from studies on vegetation rehabilitation

The profile distribution of the soil moisture is influenced by the types of vegetation rehabilitation, primarily due to the change in the land use types. In this study, different vegetation restoration types have changed the vertical profile distribution of soil moisture, which has resulted in different degrees of soil water storage deficits. Relevant results showed that the soil water deficit was serious under other vegetation restoration types with the exception of grasslands. Among them, the deep soil water deficit of PS is the most serious, while the soil water of the NG is significantly higher than that of other vegetation types, which indicates that NG may be the best type of vegetation restoration to maintain the productivity and sustainability of the ecosystem. This is consistent with previous research results^47^.

## Conclusions

By investigating the soil moisture in different types of vegetation, the research results showed the following: In the shallow layer (0–200 cm), there was no significant difference in the soil water content between the NG and the PS, but it was significantly higher than that of the PT, HR and RP. In the deep layer (200–1000 cm), the soil moisture of the NG was the highest (13.20%), which was significantly higher than that of AS, PT, PS, RP and HR. The soil water recharge of different types of vegetation varies during different periods. The main influencing factors were the characteristics of vegetation and annual rainfall. Compared with that of NG, the soil water deficit of RP and PS was the most serious, and the vegetation is the primary factor that affects the soil moisture in the deep layer. Therefore, it is recommended to choose natural recovery in the future vegetation restoration process.
